# Triple Immunochromatographic System for Simultaneous Serodiagnosis of Bovine Brucellosis, Tuberculosis, and Leukemia

**DOI:** 10.3390/bios9040115

**Published:** 2019-09-29

**Authors:** Lyubov V. Barshevskaya, Dmitriy V. Sotnikov, Anatoly V. Zherdev, Bekbolat B. Khassenov, Kayrat K. Baltin, Saule Z. Eskendirova, Kassym K. Mukanov, Kanatbek K. Mukantayev, Boris B. Dzantiev

**Affiliations:** 1 A.N. Bach Institute of Biochemistry, Research Center of Biotechnology of the Russian Academy of Sciences, Leninsky prospect 33, Moscow 119071, Russia; lyubov.barshevskaya@yandex.ru (L.V.B.); sotnikov-d-i@mail.ru (D.V.S.); zherdev@inbi.ras.ru (A.V.Z.); 2 Republican State Enterprise “National Center for Biotechnology” under the Science Committee of Ministry of Education and Science of the Republic of Kazakhstan, Nursultan 010000, Kazakhstan; khassenov@biocenter.kz (B.B.K.); baltin@biocenter.kz (K.K.B.); saule_e@mail.ru (S.Z.E.); mukanov@biocenter.kz (K.K.M.); mukantaev@biocenter.kz (K.K.M.)

**Keywords:** immunochromatographic assay, serodiagnosis, bovine, brucellosis, tuberculosis, leukemia

## Abstract

An immunochromatographic test system has been developed for the simultaneous rapid multiplex serodiagnostics of bovine brucellosis, tuberculosis, and leukemia. The test system is based on the use of a conjugate of gold nanoparticles with the chimeric protein Cysteine-A/G and three analytical zones with immobilized pathogen antigens: *Brucella abortus* lipolysaccharide, recombinant proteins MPB64 and MPB83-MPB63 of *Mycobacterium bovis*, and recombinant protein p24 of the bovine leukemia virus. Prototypes of the test system were tested on 98 samples of sera from healthy and infected animals. The diagnostic sensitivity of the developed test system was 92% for brucellosis, 92% for tuberculosis, and 96% for leukemia. False positive test results were not observed.

## 1. Introduction

The spread of infectious diseases in cattle is an urgent problem of modern animal husbandry. The diseases that inflict the highest economic damage include bovine tuberculosis, brucellosis, and leukemia [1,2,3,4]. The transmission of pathogens from infected animals to humans is also a significant hazard [1,5,6,7].

Measures to counteract and control the spread of these infections require a quick and timely diagnosis. Currently, various methods make it possible to diagnose cattle infections. The most attractive approach to mass screening studies is serodiagnosis, the detection of specific antibodies against antigens of pathogens in the blood of an animal. For numerous reasons, this approach is preferable to identifying the pathogen itself and its antigens. First, serodiagnosis uses blood serum as a universal sample for the diagnostics of any infectious diseases because the infectious process is always accompanied by an increase in the concentration of antibodies in the blood whereas a pathogen can accumulate in tissues or organs. Moreover, pathogen antigens are rapidly destroyed in the animal’s organism by means of lytic systems but antibodies can continue to circulate in the bloodstream for a long time [8,9].

To detect brucellosis, the following methods of serodiagnosis are usually used: Agglutination tests (the Rose Bengal test, the whey agglutination test, the mercaptoethanol agglutination test); the complement binding reaction [10,11,12]; the fluorescence polarization assay [13]; and ELISA [14,15]. At the beginning of their development, serological methods for the diagnostics of tuberculosis did not allow yield-adequate and accurate results; in particular, they had low specificity and sensitivity [16]. Currently, however, ELISA [17] and the fluorescence polarization assay [18] are successfully used to detect specific antibodies against *M. bovis*. Serodiagnostics of bovine leukemia virus also include modifications of ELISA [19,20], immunodiffusion in agar gel, and immunoblotting [21].

Despite these techniques’ widespread use, they have several limitations: ELISA requires the use of laboratory equipment and the involvement of qualified specialists, and agglutination methods and immunoblotting are significantly inferior in sensitivity. These limitations make it difficult to use these methods for mass screening and offsite laboratory assaying. Therefore, types of rapid non-instrumental assaying such as immunochromatography are used more widely. The immunochromatographic test strip is a multi-membrane composite mounted on a substrate. The transfer of the sample along the membranes of the test strip and the interaction of the reagents take place under the action of capillary forces. As a result, colored immune complexes are formed in specific zones of the test strip. Thus, the assay can be carried out outside the laboratory with results obtained within 10–20 min without special processing and with a simple interpretation.

Immunochromatographic tests for serodiagnosis of brucellosis in animal species have been described [22,23,24,25]. Kim et al. have developed an immunochromatographic system for the detection of specific antibodies against the bovine leukemia virus [26]. Koo et al., Sotnikov et al., and Bermúdez et al. have shown the feasibility of using immunochromatographic assays for the serodiagnosis of tuberculosis [27,28,29]. A number of commercially available kits for serodiagnosis of bovine brucellosis, tuberculosis, and leukemia are available, such as Rapid B. Brucella Ab, and Rapid B. TB Ab (BioNote Inc., Korea); Rapid Bovine Brucella Ab Test Kit (GENTAUR Molecular Products, Belgium); Brucella Rapid Test (Life Assay [PTY] LTD, South Africa); Bovine Brucella Ab Rapid Test (Quicking Biotech Co., Ltd., China); and One Step Bovine Leukemia Virus Antibody Test (Mex Biotech Hong Kong Ltd).

One rapid area of development for modern immunochromatography is the creation of multiparameter systems that allow the detection of several analytes in one test. For these purposes, several approaches have been developed that, as a rule, are based on the use of several analytical zones on one test strip. To date, multiparameter immunochromatography in veterinary serodiagnosis has been implemented only for the detection of two pathogens [30,31] and has not yet been commercialized.

Using one test system to diagnose several cattle infections simultaneously can significantly reduce not only the assay duration but also the expenditure of reagents and consumables [32]. Our study’s aim was to develop a multiparameter immunochromatographic test system for the simultaneous detection of specific antibodies against three pathogens of priority diseases of cattle: *Brucella abortus, Mycobacterium bovis*, and bovine leukemia virus (BLV).

## 2. Materials and Methods

### 2.1. Preparing Gold Nanoparticles

Nanoparticles were synthesized by the Frens method [33]. One ml of a 1% aqueous solution of HAuCl_4_ (Sigma-Aldrich, USA) was added to 100 mL of water, with the mixture heated to boiling; then, 1.5 mL of a 1% aqueous solution of sodium citrate was added with vigorous stirring. The mixture was boiled for 15 min then cooled and stored at +4 °C.

### 2.2. Determination of the Size of the Obtained Gold Nanoparticles by Transmission Microscopy

Pictures of colloidal gold preparations were obtained using a CX-100 transmission electron microscope (Jeol, Japan) at an accelerating voltage of 80 kV and a magnification of 33,000×. Photos were scanned and analyzed using the Image Tool program (UTHSCSA, USA), with characterization of at least 120 images of single particles.

### 2.3. Obtaining Conjugates of Gold Nanoparticles with Cysteine–A/G Protein

Conjugation was performed according to the procedure described in [23], with minor modifications. A solution of gold nanoparticles (optical density at 520 nm: OD_520_ = 1) was adjusted with potassium carbonate to pH 8.5–9.0 (basing the results of our previous studies [34,35]), after which the Cys-A/G protein (ProsPec, Israel) was added to this solution being diluted to the concentration of 15 μg/mL. The mixture was incubated for 1 h at room temperature; then, a 10% BSA solution (Boval Biosolutions, USA) was added and the mixture kept for another 10 min with vigorous stirring. The resulting solution was centrifuged for 15 min at 10,000× *g*. The precipitate was collected, and a 10% BSA solution was added to 1 mL. The resulting solution was characterized by measuring optical density at 520 nm and stored at 4 °C.

### 2.4. Preparations of Antigens of Brucella abortus, Mycobacterium bovis, and BLV

The antigens used in the present work—LPS of *Brucella abortus* and p24 of BLV—were provided by the National Center for Biotechnology of the Republic of Kazakhstan (Nursultan, Kazakhstan). Recombinant protein MPB64 of *Mycobacterium bovis* was provided by S.F. Biketov (State Research Center for Applied Microbiology and Biotechnology, Obolensk, Russia). Recombinant fusion protein MPB83-MPB63 was provided by D.V. Kolibo (A.V. Palladin Institute of Biochemistry, Kiyiv, Ukraine) [36].

### 2.5. Making of Immunochromatographic Test Systems

The complete set of test strips included a working nitrocellulose membrane, membranes for application of a sample and conjugate, and a final absorbent membrane. To make the test systems, we used an mdi Easypack CNPH90 working membrane (Advanced Microdevices, India) and a CFSP 223000 absorbent membrane (Millipore, MA, USA), which we also used as a sample application membrane. An untreated PT-R5 mdi membrane (Advanced Microdevices, India) was used as a conjugate application membrane. Using an IsoFlow automatic dispenser (Imagene Technology, NH, USA), we formed three analytical zones on a working nitrocellulose membrane; immobilized in them were (i) LPS preparations (0.5 mg/mL) in Na-carbonate buffer (pH 9.2); (ii) the mixture of MPB64 (0.5 mg/mL) and MPB83-MPB63 (1 mg/mL) in phosphate-buffered saline (pH 7.4); and (iii) p24 (1 mg/mL) in phosphate-buffered saline (pH 7.4). A preparation of 2 μL per 1 cm of the strip was applied. For some lots of test strips, a control zone with bovine immunoglobulins was also applied on a working membrane. Conjugates of gold nanoparticles with Cys-A/G protein (D_520_ = 10, application volume of 13 μL per 1 mm) were applied to the substrate for the conjugate. 

After application of the reagents, the membranes were air-dried at 20–22 °C for at least 20 h. A multi-membrane composite was assembled, which was cut into 3.5 mm wide strips using an Index Cutter-1 automatic guillotine cutter (A-Point Technologies, NJ, USA). Cutting and packaging were carried out at 20–22 °C in a special room with a relative humidity of not more than 30%. Packed test strips were stored at 20–22 °C.

### 2.6. Serum Panels

The serum samples of cows were collected in the course of previous investigations of the authors [23,24,25,27]. Blood serum were obtained from the “Russian Black Pied” and ‘Red Steppe’ cattle breeds aged from 1 to 4 years and weighing from 200 to 450 kg, “Kazakh Whiteheaded” cattle breed mostly at the age of 2 years and weighing from 200 to 550 kg.

The given study was based on the stored panels of serum and did not involve experiments with animals. A characterized panel of blood serum of cows infected with *Br. abortus* and bovine leucosis virus, as well as healthy animals, was provided by the National Center for Biotechnology of the Republic of Kazakhstan (Nursultan, Kazakhstan). A panel of seropositive serum to *M. bovis* was created at the Research Center of Biotechnology of the Russian Academy of Sciences (Moscow, Russia).

### 2.7. ELISA Detection of Antibodies against LPS of Br. abortus, MRT64 of M. bovis, and p24 of BLV in bovine serum

Sorption of antigen preparations in the wells of a 96-well microplate was performed overnight at 4 °C from 100 μL of a solution with the antigen concentration of 1 μg/mL in 50 mM carbonate buffer, pH 9.6. The microplate was rinsed four times with 50 mM K-phosphate buffer, pH 7.4, comprising 0.1 M NaCl and 0.05% Triton X-100 (PBST), after which 100 μL of sera diluted with PBST from 1:10^2^ to 1:10^5^ in increments of two were added to the wells; and the mixture was incubated for 1 h at 37 °C. Then the microplate was rinsed again, 100 μL of solution of anti-bovine IgG monoclonal antibodies labeled with horseradish peroxidase (IMTEC, Russia) at the concentration of 160 ng/mL in PBST was added, and the mixture was incubated for 1 h at 37 °C. After rinsing the microplate (three times with PBST and once with distilled water), we determined the peroxidase activity of the enzyme label bound to the carrier. To do this, we added 100 μL of a 0.4 mM solution of the substrate 3,3´,5,5´–tetramethylbenzidine (TMB, Sigma, USA) comprising 0.01% H_2_O_2_ in 30 mM citrate buffer (pH 4.0) to the wells, incubated the mixture for 15 min at room temperature, and measured the absorbance at 450 nm (OD_450_) on a Zenyth 3100 plate photometer (Anthos Labtec Instruments, Austria).

### 2.8. Immunochromatographic Assay

ICA was performed at room temperature. 1 μL of blood serum was added to an Eppendorf tube, followed by three drops (100 μL) of PBS containing 1% Tween-20, and the test strip was placed vertically into the Eppendorf tube. After 10 min, the result of ICA was visually evaluated.

## 3. Results and Discussion

### 3.1. Characterization of Gold Nanoparticles

**Figure 1:**
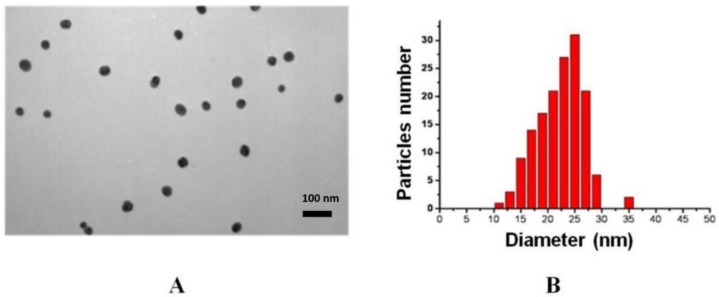
Micrograph of the preparation of gold nanoparticles obtained by transmission electron microscopy—enlargement 33,000× (**A**). Histogram of the distribution of nanoparticles by diameter (**B**).

Gold nanoparticles were synthesized by the Frens method [33], which makes it possible to obtain particles of a certain size depending on the amount of added reducing agent. The optimal size of gold nanoparticles for immunochromatography is 20–40 nm [37]. Transmission electron microscopy was used to determine the average diameter and homogeneity of the preparations obtained. Figure 1 shows a micrograph of gold nanoparticles as well as a histogram of the distribution of particles by diameter. According to the data obtained, the synthesized nanoparticles are characterized by a spherical shape and a homogeneous distribution by size. The length of the major axis was 24 ± 5 nm; the length of the smaller axis was 20 ± 4 nm. The average diameter of gold nanoparticles was 22 ± 5 nm.

### 3.2. Testing of bovine serum by ELISA

To confirm the diagnosis put forward (i.e., leukemia, brucellosis, or tuberculosis), to determine antibody titers, and to establish a cutoff level between infected and healthy animals, an additional testing by ELISA was performed on a panel containing 98 samples (52 positive samples for leukemia, 12 positive samples for tuberculosis, 13 positive samples for brucellosis, and 21 serums of healthy animals).

**Figure 2:**
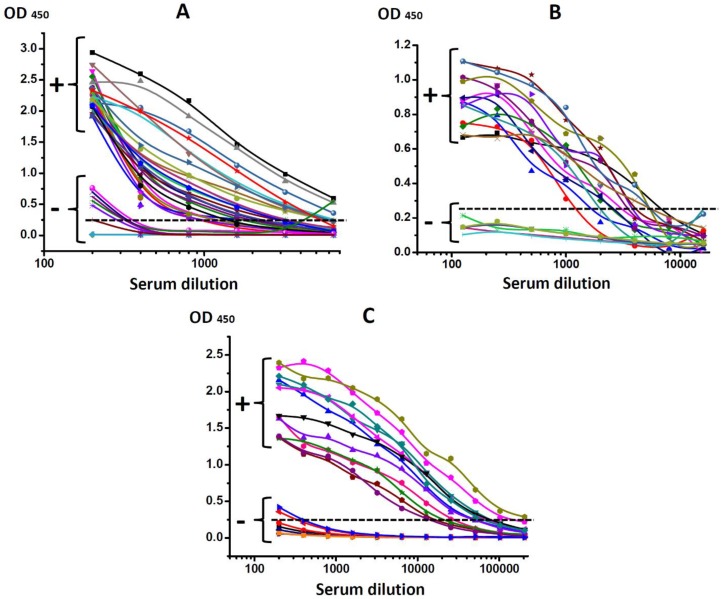
Testing of bovine sera obtained from healthy (−) and infected animals (+) diagnosed with (**A**) leukemia (BLV); (**B**) brucellosis (*Br. abortus)*; (**C**) tuberculosis (*M. bovis*).

The initial diagnosis was confirmed according to the results of ELISA testing of all infected animals. The titers of antisera were stated as maximal dilution, giving a signal to noise ratio of >3. For serodiagnosis of bovine leukemia, the threshold titer for distinguishing positive and negative samples was 1:400 (Figure 2A), for serodiagnosis of bovine brucellosis—1:500 (Figure 2B), and for serodiagnosis of bovine tuberculosis—1:800 (Figure 2C).

### 3.3. Choice of Format of Immunochromatographic Serodiagnosis and Making of Test Systems

Currently, several formats of immunochromatographic serodiagnosis are known. One involves the use of a sandwich complex of antigens to detect the target analyte. For instance, Huang et al. developed a test for the simultaneous diagnostics of two equine diseases caused by *Babesia caballi* and *B. equi* [30]. They used an approach based on the use of the property of polyvalency of antibodies and two pairs of antigenic preparations, labeled with a marker and immobilized in the analytical zone of the test (each pair for one pathogen). Kim et al. used the same approach for the diagnostics of bovine babesiosis caused by *B. bovis* and *B. bigemina* [31].

For the multiplex serodiagnosis of bovine brucellosis, tuberculosis, and leukemia, we chose a scheme that uses a conjugate of gold nanoparticles with Cys-A/G protein which immobilizes the antigens of *Br. abortus, M. bovis*, and BLV in the analytical zone (each in its own section of the test strip). This scheme assumes the interaction of the marker conjugate with all immunoglobulins in the sample and the subsequent detection of specific immunoglobulins by binding them in one of the analytical zone’s three sections. As a result of affine interactions (in the presence of specific antibodies in the sample) in one of the sections of the analytical zone, the Cys-A/G protein–antibody–antigen complexes are formed; they are visualized as colored bands.

Such an immunochromatography scheme makes it possible to use the same conjugate of a marker with an immunoglobulin-binding protein to diagnose all three diseases. Using the recombinant chimeric protein Cys-A/G allows a high density of immunoglobulin-binding sites to be achieved on the conjugate of gold nanoparticles as well as combining the specificity of proteins A and G to different classes of immunoglobulins in one conjugate [38,39]. In addition, the high affinity of gold for sulfur-containing groups enables the cysteine fragment of the protein to obtain a stable conjugate that retains activity for a long time.

The optimal conditions for immunochromatographic serodiagnosis of cattle diseases were determined, at first, in the form of individual tests for one disease. When optimizing the mono tests, we used the variants of immobilization of reagents on the working membrane (presented in Table 1). For example, the concentrations of used preparations of the antigens of *Br. abortus, M. bovis*, and BLV varied from 0.2 to 2 mg/mL; the working range of the concentrations of applied reagents was 0.5–1 mg/mL. Out of immunoglobulin-binding proteins (Protein A; Protein G; rabbit anti-bovine IgG; Cysteine-A/G), we chose the Cysteine-A/G protein for conjugation with gold nanoparticles at the concentration of 15 μg/mL when conjugated.

Before testing, we diluted sera 100 times in accordance with recommendations given in [38]. The need for such a degree of sera dilution is a consequence of a more than ten-fold excess of antibodies concentration as compared with the binding ability of the immunoglobulin-binding protein molecules immobilized on the surface of gold nanoparticles. As a result, when undiluted or slightly diluted serum is used, most antibodies do not bind to gold nanoparticle conjugate, and free and labeled antibodies compete for interaction with the antigen in the test zone, resulting in a decrease in the recorded signal.

The selected parameters (see allocations in Table 1) were used to assemble a multiplex system for detecting antibodies to the pathogens of bovine brucellosis, tuberculosis, and leukemia. We used gold nanoparticles as markers in the test system because this marker makes it possible to visually register the assay results. In addition, the process of preparation of gold nanoparticles is inexpensive and easily scalable and is not labor intensive. Gold nanoparticles do not undergo modifications and do not lose their properties for a long time.

We implemented a direct assay scheme with the immobilization of antigen in the analytical zone of the test strip and the conjugation of a nanodispersed marker with an immunoglobulin-binding reagent, ensuring the test system’s high sensitivity and specificity. A comparison of the membrane carriers showed the optimal use of a working nitrocellulose membrane of the type 90CNPH (Advanced Microdevices, Haryana, India). As a membrane for the sample application and the final absorbent membrane, we chose the CFSP 223000 membrane (Millipore, MA, USA) for its high absorbency and mechanical strength. For the application of a conjugate of gold nanoparticles with Cys-A/G protein, we chose the PT-R5 membrane. 

### 3.4. Testing of Prepared Test Systems on Pathogenic Material

Testing of the developed test system was carried out using sera preliminarily characterized by ELISA. The serum panel consisted of 98 samples, including 52 positive samples for leukemia, 12 positive samples for tuberculosis, 13 positive samples for brucellosis, and 21 bovine sera with a negative reaction to leukemia, tuberculosis, and brucellosis.

The test system was characterized by the sensitivity parameter on positive sera from the biosample panel. The data obtained are presented in Table 2. According to the test results, 12 out of 13 brucellosis sera, 11 out of 12 tuberculosis sera, and 50 out of 52 leukemia sera were found positive. Thus, the test system’s diagnostic sensitivity was 92% for brucellosis, 92% for tuberculosis, and 96% for leukemia.

**Figure 3:**
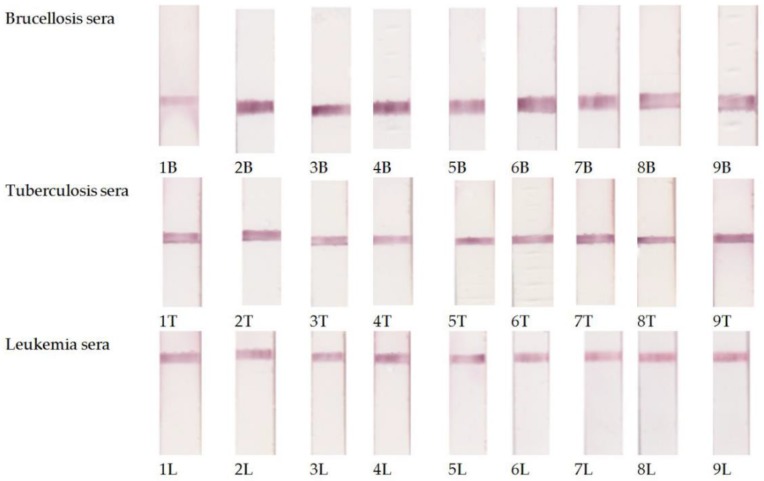
Examples of testing of the serum panel preliminarily characterized by ELISA using the developed multiplex immunochromatographic system.

**Figure 4:**
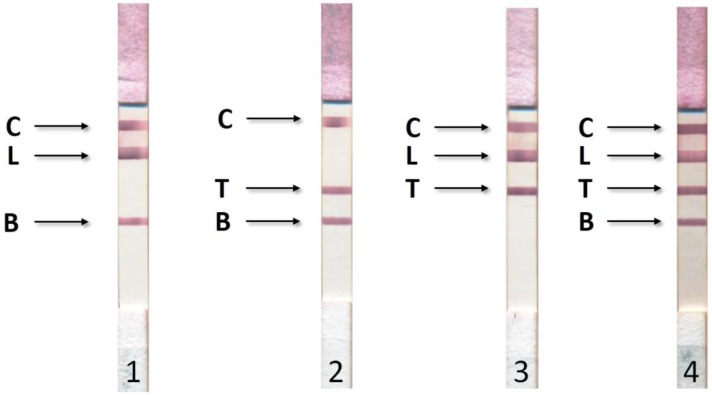
Results of ICA for simultaneous detection of bovine brucellosis (B), tuberculosis (T), and leukemia (L). **1**—positive results for leukemia and tuberculosis; **2**—positive results for leukemia and brucellosis; **3**—positive results for tuberculosis and brucellosis; **4**—positive results for leukemia, tuberculosis, and brucellosis. C—control line with immobilized rabbit anti-bovine immunoglobulins.

Figure 3 shows the photographs of the working membranes of test systems after assaying 27 sera (9 for each disease). In any of the tested sera, we detected no more than one disease. When we combined the sera, we observed the corresponding staining of several analytical zones (Figure 4).

A panel of 21 animal sera with no signs of brucellosis, tuberculosis, and leukemia was tested. ELISA confirmed the absence of antibodies against the antigens of *Br. abortus, M. bovis*, and BLV in the sera. None of the sera yielded a positive result. Thus, the specificity of ICA-diagnostics was 100% for all three pathogens.

## 4. Conclusions

A multiplex immunochromatographic test system for the simultaneous serodiagnosis of bovine brucellosis, tuberculosis, and leukemia was developed and tested. The results of the testing demonstrate the possibility of the simultaneous detection of specific antibodies to pathogens of three cattle diseases with a high sensitivity (92–96%) and specificity (100%). The duration of the testing was 10 min.

When separate tests are used to diagnose each disease, the cost of assaying increases in proportion to the number of pathogens. In the case of the multiplex immunochromatographic serodiagnosis, an increase in the number of diagnosed diseases involves only the addition of an appropriate number of analytical zones with immobilized antigens. The membrane set and the marker conjugate expenditure remain unchanged. This provides significant economic advantages to multiplex immunochromatographic systems compared with monotests. In addition, the use of multitests leads to an increase in the assay productivity and a reduction in the biosample usage and the number of manipulation stages, which is important when working with infectious material.

## Figures and Tables

**Table 1:** Optimization of ICA conditions (selected parameters are highlighted (red) in color).

Parameter								
Immunoglobulin-Binding Protein	Protein A	Protein G	Cysteine–A/G Protein		Rabbit Anti-Bovine IgG
Concentration of protein upon conjugation (µg/mL)	5	10	15		20
Solutions for application of antigens	20 mM Na-citrate (pH 6.0)	20 mM PBS (pH 7.4)*	10 mM Tris-HCl (pH 7.5)	20 mM HEPES (pH 7.6)	10 mM Na-carbonate (pH 9.2)**
Concentration of LPS of *Br. abortus* (mg/mL)	0.2	0.5	1		2
Concentration of МРB64 (mg/mL)	0.2	0.5	1		2
Concentration of MPB83-MPB63 (mg/mL)	0.2	0.5	1		2
Concentration of p24 BLV (mg/mL)	0.2	0.5	1		2
Type of working membrane	CNPF10	CNPC5	CNPC12	CNPC15	90CNPH

* - for proteins, ** - for LPS.

**Table 2:** Results of immunochromatographic testing of bovine sera.

	Brucellosis			Tuberculosis			Leukemia		
	+	-	Total	+	-	Total	+	-	Total
Positive serum	12	1	13	11	1	12	50	2	52
Negative serum	0	21	21	0	21	21	0	21	21
Total			34			33			73
